# Supplementary material for: The causal role of circulating inflammatory markers in osteoporosis: a bidirectional Mendelian randomized study
Source: Front Immunol. 2024 Jul 18;15:1412298. doi: 10.3389/fimmu.2024.1412298 (PMC11291241; doi:10.3389/fimmu.2024.1412298)
Supplement: Supplementary file 1 [file DataSheet_1.zip › supplementary material/OSTEOPOROSIS-CIM/GCST90274833.tsv.gz_forest.pdf]

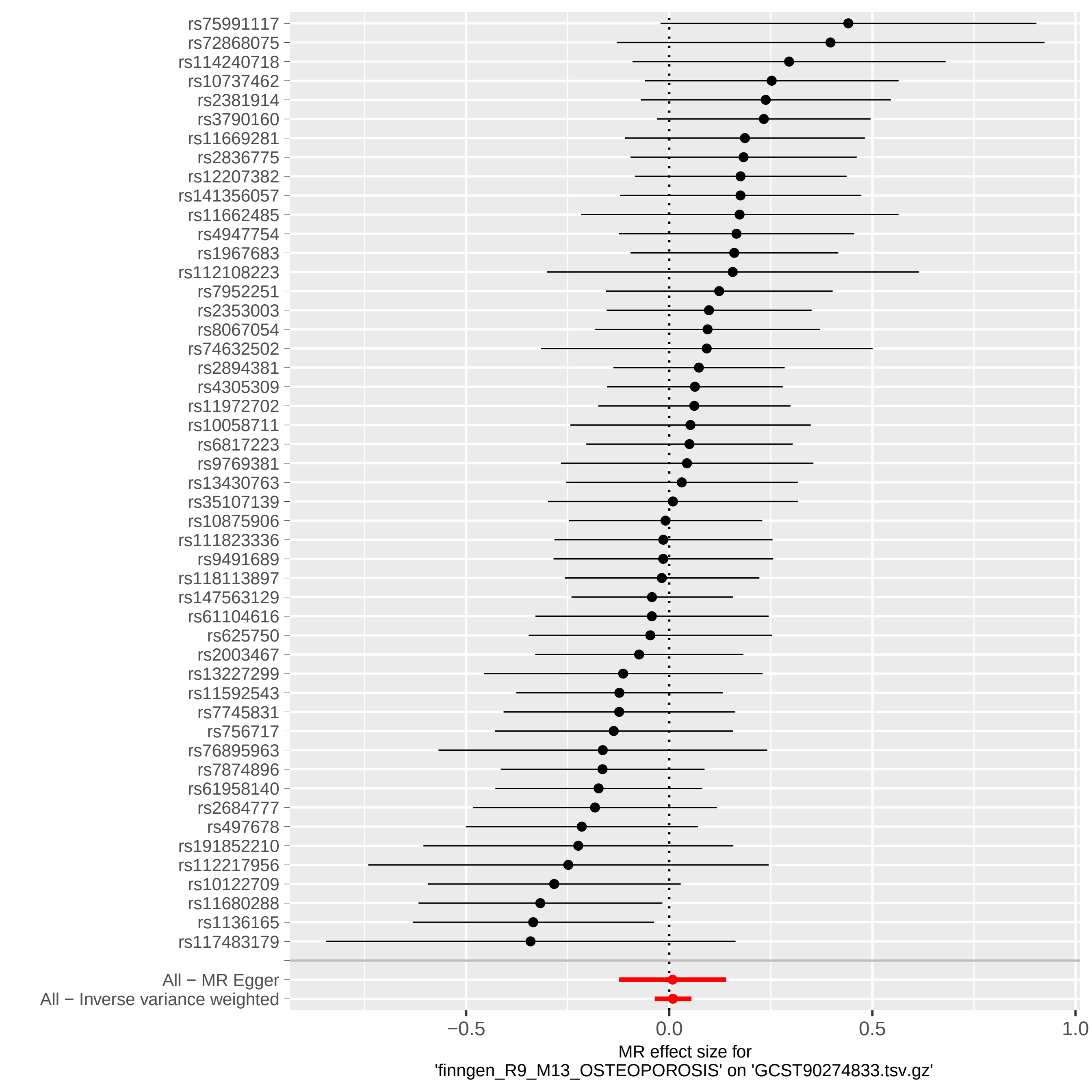
rs75991117
rs72868075
rs114240718
rs10737462
rs2381914
rs3790160
rs11669281
rs2836775
rs12207382
rs141356057
rs11662485
rs4947754
rs1967683
rs112108223
rs7952251
rs2353003
rs8067054
rs74632502
rs2894381
rs4305309
rs11972702
rs10058711
rs6817223
rs9769381
rs13430763
rs35107139
rs10875906
rs111823336
rs9491689
rs118113897
rs147563129
rs61104616
rs625750
rs2003467
rs13227299
rs11592543
rs7745831
rs756717
rs76895963
rs7874896
rs61958140
rs2684777
rs497678
rs191852210
rs112217956
rs10122709
rs11680288
rs1136165
rs117483179
All − MR Egger
All − Inverse variance weighted
−0.5
0.0
0.5
1.0
MR effect size for
'finngen_R9_M13_OSTEOPOROSIS' on 'GCST90274833.tsv.gz'
